# Drug-Coated Balloon-Only Angioplasty Outcomes in Diabetic and Nondiabetic Patients with De Novo Small Coronary Vessels Disease

**DOI:** 10.1155/2021/2632343

**Published:** 2021-12-01

**Authors:** Botey Katamu Benjamin, Wenjie Lu, Zhanying Han, Liang Pan, Xi Wang, Xiaofei Qin, Guoju Sun, Xule Wang, Yingguang Shan, Ran Li, Xiaolin Zheng, Wencai Zhang, Qiangwei Shi, Shuai Zhou, Sen Guo, Peng Qin, Chhatra Pratap Singh, Jianzeng Dong, Chunguang Qiu

**Affiliations:** ^1^Department of Cardiovascular Medicine, The First Affiliated Hospital of Zhengzhou University, Zhengzhou, China; ^2^Department of Geriatric Cardiology, The First Affiliated Hospital of Zhengzhou University, Zhengzhou, China

## Abstract

**Background:**

The revascularization of small vessels using drug-eluting stents remains challenging. The use of the drug-coated balloon is an attractive therapeutic strategy in de novo lesions in small coronary vessels, particularly in the diabetic group. This study aimed to assess the outcomes of DCB-only angioplasty in small vessel disease.

**Methods:**

A total of 1198 patients with small vessel disease treated with DCB-only strategy were followed. Patients were divided into the diabetic and nondiabetic groups. Clinical and angiographical follow-up were organized at 12 months. The primary endpoints were target lesion failure and secondary major adverse cardiac events.

**Results:**

There was a significantly higher rate of target lesion failure among diabetic patients compared to nondiabetic [17 (3.9%) vs. 11 (1.4%), *P*=0.006], taken separately, the rate of target lesion revascularization significantly differed between groups with a higher rate observed in the diabetic group [9 (2%) vs. 4 (0.5%), *P*=0.014]. Diabetes mellitus remained an independent predictor for TLF (HR: 2.712, CI: 1.254–5.864, *P*=0.011) and target lesion revascularization (HR: 3.698, CI: 1.112–12.298, *P*=0.033) after adjustment. However, no significant differences were observed between groups regarding the target vessel myocardial infarction (0.6% vs. 0.1%, *P*=0.110) and MACE [19 (4.4%) vs. 21 (2.7%), *P*=0.120].

**Conclusion:**

Drug-coated balloon-only treatment achieved lower incidence rates of TLF and MACE. Diabetes is an independent predictor for target lesion failure and target lesion revascularization at one year following DCB treatment in small coronary vessels. We observed no significant differences between groups regarding MACE in one year.

## 1. Introduction

Small vessel coronary disease is an independent predictor for poor outcomes after the percutaneous coronary intervention (PCI) and accounts each year for 30 to 50% of all-comers coronary intervention procedures worldwide [1–4]. The best treatment strategy for small vessel coronary disease remains a real challenge [5]. Small vessel disease (SVD) poses concrete limitations to the use of drug-eluting stents (DES); the implantation of DES in small vessels is confronted with higher rates of stent failure, restenosis, and repeat revascularization consecutive to the late lumen loss (LLL) [3–8]. This is partly related to the fact that DES leave permanent metallic struts and polymeric matrix within treated vessels which behave as persistent inflammatory stimulus and hinder the full restoration of vessels endothelial functions and the capacity to increase lumen diameter over time [9, 10]. The accelerated LLL is related to the small vessel's restricted capability to accommodate to even small neointimal proliferation following stent implantation; that is, for a given neointimal proliferation, the relative lumen reduction will be more pronounced in the small vessel as compared to big vessel [1, 11–13]. Besides, there is an established association between small vessel coronary disease with diabetes mellitus, which is also a powerful predictor for poor outcomes after PCI [1, 14–20]. Conceived to overcome the drawbacks observed with DES, emerging evidence suggests using drug-coating balloons (DCB) as an interesting alternative treatment strategy in small vessel disease [21–25]. Because of the higher prevalence of SVD observed in diabetic patients, this group of patients is more likely to be treated with the drug-coated balloon. There are no available data comparing DCB outcomes in diabetic and nondiabetic patients with SVD to the best of our knowledge. It is not clear whether treatment strategy with DCB in this particular setting will result in the same trend of outcomes as observed when DES are used provided that DCB fulfill therapeutic goals of DES without reproducing its limitations and drawbacks. Therefore, this study aims to assess diabetic and nondiabetic patients' outcomes after drug-coated balloon-only strategy in de novo small vessel coronary disease.

## 2. Material and Methods

### 2.1. Study Design and Population

This is a prospective, single-center, observational study of 1198 patients undergoing PCI for de novo lesion in the native small coronary vessels with DCB as the only therapeutic strategy. We defined small vessel disease as reference vessel diameter ≤2.75 mm. Based on the presence of diabetes, patients were divided into the diabetic and nondiabetic groups. We considered as diabetic patients with fasting plasma glucose ≥126 mg/dL (7.0 mmol/L) and/or two-hour plasma glucose ≥200 mg/dL (11.1 mmol/L) during an oral glucose tolerance test and/or HbA1c ≥ 6.5% [26]. Clinical follow-up was conducted at 12-month postprocedure or at any time when needed.

### 2.2. Exclusion Criteria

A larger vessel with >2.75 mm diameter, in-stent restenosis, hybrid treatment of the target lesion, simultaneous treatment of small and large vessels lesions, simultaneous treatment of in-stent restenosis and de novo lesions, renal failure requiring dialysis, contraindication to dual antiplatelet therapy, and bailout stent implantation.

### 2.3. Study Endpoints and Definition

The primary endpoint of the study were target lesion failure (TLF) defined as the composite of ischemia-driven revascularization of the target lesion, myocardial infarction related to the target vessel, or cardiac death related to the target vessel. Whenever it was not possible to determine with certainty whether the myocardial infarction or death was related to the target vessel, it was considered a TLF. The secondary endpoints were major adverse cardiac events (MACE) defined as the composite of all-cause death, myocardial infarction, and target vessel revascularization (TVR). We considered as clinically driven target lesion revascularization (TLR) any revascularization of the target lesion performed due to a stenosis >50% and either evidence of clinical or functional ischemia or recurrence of the clinical syndrome for which the initial procedure was performed [27]. TLR was defined as any repeat percutaneous coronary intervention of the target lesion (including 10 mm proximal and 10 mm distal to the index device) or bypass surgery of the target vessel motivated by restenosis or other complications [27]. Target vessel myocardial infarction was defined as any myocardial infarction related to the target vessel or when not clearly related to any other vessel [27]. The diagnosis of myocardial infarction was made when the following criteria were met: detection of increase and or decrease of cardiac biomarkers levels; at least one value above the 99th percentile of the upper reference limit, together with evidence of myocardial ischemia, as recognized by at least one of the following: symptoms of ischemia, electrocardiogram changes of new ischemia or development of pathologic Q waves, and imaging evidence of new loss of viable myocardium or new regional wall motion abnormality [28]. Cardiac death was defined as death resulting from cardiovascular causes such as death caused by acute myocardial infarction, sudden cardiac death, including unwitnessed death, heart failure, stroke, cardiovascular procedures, cardiovascular hemorrhage, and death resulting from other cardiovascular causes [27]. We considered all-cause death as death related to any possible cause and TVR as any repeat percutaneous intervention or surgical bypass of any segment of the target vessel.

### 2.4. Interventions

We included patients whose interventions and postprocedure period were managed in line with our established criteria. Patients received oral aspirin 300 mg at least 24 h before the intervention, oral clopidogrel or ticagrelor at least six hours before the procedure, respectively, at 300 mg and 180 mg (except for stable patients on ongoing dual antiplatelet therapy since a certain time) and then maintained aspirin at 100 mg/day, clopidogrel at 75 mg/day, or ticagrelor 90 mg twice daily. In patients with the acute coronary syndrome, a loading dose of aspirin 300 mg and ticagrelor 180 mg or clopidogrel 600 mg were orally administered at the admission before the procedure. The choice between clopidogrel and ticagrelor was at the physician's discretion. Intravenous administration of unfractionated heparin at 100 IU/kg was done a few minutes before PCI and activated clotting time was maintained at 250 to 350 s throughout the intervention. Careful lesion preparation was observed before applying the DCB and balloons were not used for postdilation once the DCB was deployed; the choice of the appropriate technique for lesion preparation and balloon selection was at the operator's discretion. We regarded as successful intervention treatment all postprocedural visually estimated ≤30% residual stenosis after PCI. After the procedure, patients received a dual antiplatelet therapy (DAPT) regimen, including daily aspirin 100 mg in association with clopidogrel 75 mg or ticagrelor 90 mg twice daily for at least 6 months.

### 2.5. Device Description

All patients were treated with the SeQuent Please which is a paclitaxel-coated balloon catheter (B. Braun Melsungen, Germany). The paclitaxel is coated at a dose of 3 *μ*g/mm^2^ balloon surface, a matrix coating made of paclitaxel plus hydrophilic spacer (iopromide). A minimum of 40 seconds inflation time is needed to allow sufficient drug to be released into the vessel wall; only 4.5% of the drug remains on the balloon [4].

### 2.6. Quantitative Coronary Angiography

Quantitative coronary angiography measurements were performed off-line using a validated edge detection system (QAngio XA V7.3, Medis Medical Imaging, Netherlands) at a core laboratory (The First Affiliated Hospital of Zhengzhou University, Zhengzhou, China) by a team of experienced personnel who were blinded.

### 2.7. Follow-Up

All patients were clinically followed up at 12 months (visits in the hospital and by telephone); we used a structured clinical questionnaire to assess medication, quality of life, and events. The angiographic follow-up was organized at 12 months or at any time when needed. Angiographic data were vouched by a blinded core lab, and an independent clinical committee adjudicated all the study endpoints.

### 2.8. Statistical Analysis

Categorical variables are expressed as frequencies and percentages while continuous variables are reported as mean ± standard deviation. As appropriate comparisons between categorical variables were evaluated using a 2-tailed Fisher's exact test or Pearson's chi-squared test, continuous variables were compared using Student's *t* test. To identify independent predictors of TLF and TLR, a Cox proportional hazard multivariate analysis was performed to calculate the hazard ratios (HR) with 95% confidence intervals (CI). Statistical significance was accepted for bilateral *P* < 0.05.

**Figure 1:**
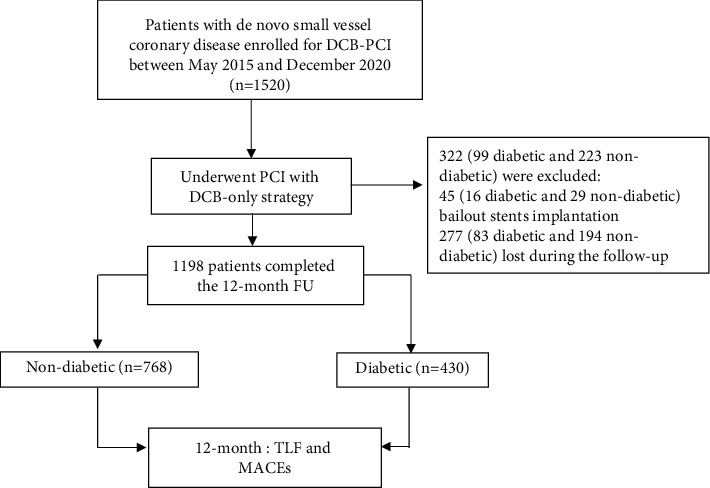
Study diagram. DCB: drug-coated balloon; FU: follow-up; MACEs: major adverse cardiac events; PCI: percutaneous coronary intervention; TLF: target lesion failure.

The summary of the method is presented in Figure 1.

## 3. Results

### 3.1. Patients Baseline Characteristics

From a total of 1198 patients, 831 were male, and 367 were female; 430 had diabetes, and 768 were nondiabetic. Diabetic patients were significantly older and had a significantly higher proportion of hypertension, hyperlipidemia, and renal failure compared to the nondiabetic. Data are presented in Table 1.

### 3.2. Baseline Lesions Characteristics

Of 1351 treated lesions, 483 were observed in the diabetic group versus 868 in the nondiabetic group. There were numerically higher proportion of bifurcation, ostial lesion, thrombosis, and diffused lesion in the diabetic group, without significant differences. There were a numerically higher proportion of chronic total occlusion, calcified lesions, and longer mean lesion length in the nondiabetic group compared to the diabetic. Data are presented in Table 2.

### 3.3. Procedure Characteristics

Comparable results were observed between groups in terms of the reference vessel diameter (2.5 ± 0.2 vs. 2.5 ± 0.8; *P*=0.366), DCB inflation pressure (8.3 ± 5.12 atm vs. 8.1 ± 1.42 atm; *P*=0.416), DCB inflation time (59.9 ± 3.76 s vs. 60.1 ± 4.93 s; *P*=0.474), and postprocedural mean diameter residual stenosis (20.3 ± 11.4 vs. 19.3 ± 11.6; *P*=0.145) in the diabetic and nondiabetic, respectively. Data are provided in Table 3.

### 3.4. Outcomes

**Figure 2:**
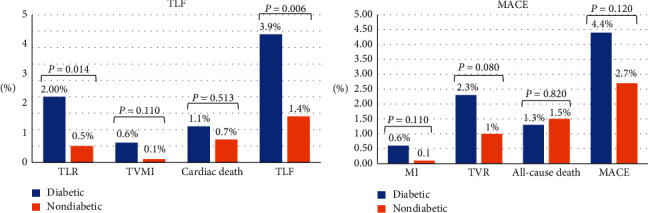
Study endpoints at 12 months. MACE: major adverse cardiac events; MI: myocardial infarction; TLF: target lesion failure; TLR: target lesion revascularization; TVMI: target vessel myocardial infarction; TVR: target vessel revascularization.

**Figure 3:**
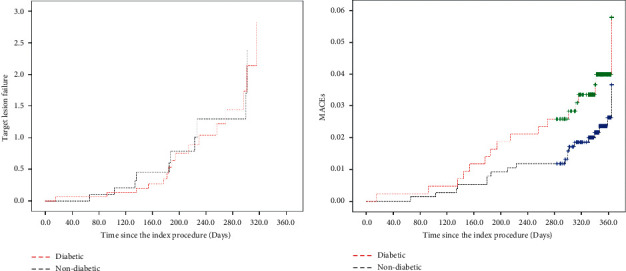
Kaplan–Meier curves of the TLF and MACE at 12 months. MACEs: major adverse cardiac events; TLF: target lesion failure.

Of 1198 enrolled patients, 535 (44.65%) had angiographic follow-up at 1 year. We observed significant differences between the groups regarding minimum lumen diameter (MLD) (1.89 ± 0.53 vs. 2 ± 0.39; *P*=0.07), LLL (0.17 ± 0.37 vs. 0.09 ± 0.25; *P*=0.003), and binary restenosis (4.8% vs. 1.1%; *P*=0.008) in the diabetic and nondiabetic, respectively. There were significant differences between groups in terms of TLF; the higher incidence rates were observed in the diabetic group (3.9% vs. 1.4%; *P*=0.006). TLF components taken separately showed significantly higher rates of TLR in the diabetic group (2% vs. 0.5%; *P*=0.014). However, comparable results were observed between groups regarding the target vessel myocardial infarction (0.6% vs. 0.1%; *P*=0.110). There were no significant differences between the two groups in the MACE (4.4% vs. 2.7%; *P*=0.120). Data are presented in Table 4 and Figures 2 and 3.

### 3.5. Independent Predictor of Target Lesion Failure and Target Lesion Revascularization

We performed the multivariate logistic regression analysis to identify independent predictors of TLF and TLR; diabetes remained an independent predictor for TLF and MACE after adjustment. Results are presented in Table 5.

## 4. Discussion

In keeping with the proposed appropriate cutoff to define small vessels, and with the majority of DCB studies on the small vessel that used <2.8 mm as the cutoff point for small vessel definition, we defined small vessel as estimated reference vessel diameter ≤2.75 mm [4, 7]. Many trials have demonstrated the noninferiority and safety of DCB compared to DES in treating the small coronary vessel [23, 29, 30]. This is the first clinical trial to compare the DCB outcomes in diabetic and nondiabetic patients with de novo small coronary vessels. The main findings of this prospective single-center observational study indicate the significantly higher rates of TLF (3.9% vs. 1.4%; *P*=0.006) and TLR (2% vs. 0.5%; *P*=0.014) in the diabetic group. A higher MACE rate (4.4% vs. 2.7%; *P*=0.120) was observed with diabetic arm without reaching statistical significance. Diabetes remained an independent predictor for TLF (HR: 2.712; CI: 1.254–5.864; *P*=0.011) and TLR (HR: 3.698; CI: 1.112–12.298; *P*=0.033) after adjustment. Our findings are in keeping with the previous studies that reported acceptable low rates of TLF and MACE with DCB in the small vessel [11, 21, 29, 31–37]. The PEPCAD I study reported 6.1% MACE events' rates after 12-month follow-up; the BASKET-SMALL 2 trial observed 7.5% MACE rates in the DCB arm [23, 31]. The DCB angioplasty in elderly patients with SVD reported a 9-month MACE of 4.2% events rates [32]. The RESTORE SVD China Randomized trial reported 4.3% TLF events' rates [30]. We observed the lowest TLF (2.3%) and MACE events' rates (3.3%) compared to these cited studies. The trend toward lower MACE rates consistently observed with the use of drug-coated balloons in SVD is related to the “leave nothing behind strategy.” With the DCB-only angioplasty strategy, it has been observed a postprocedure luminal enlargement; the test using acetylcholine revealed a less pronounced coronary endothelial dysfunction and the absence of acute or late thrombosis [4]. We observed lower MACE rates even in the diabetic group as compared to other studies. Megaly et al. found that DCB had a trend toward lowering TLR in diabetic patients [33]. The BELLO study found that the significant benefit of DCB in the small vessels holds even in the high-risk diabetic patients [29].

The lowest MACE incidence in our study compared to other studies may be partly related to the sample size differences; all the cited studies had a smaller sample size compared to our study. Second, different inclusion criteria were considered; Sinega et al., for instance, enrolled uniquely elderly people who are more likely to have a higher proportion of all the classical risk factors for MACE compared to the general population. DCB angioplasty followed by bailout BMS implantation is known as an independent predictor for poor outcomes [11, 33, 34]. All the bailout stenting was excluded from our study, contrary to the mentioned studies. Besides, appropriate lesion preparation is the key for a successful DCB angioplasty; we were particularly careful to include only procedures where the established recommendations for lesion preparation were observed [38]. Our findings corroborate the results of the trial by Jalaluddin, which reported significantly higher rates of TLR (1.4% vs. 0.6%; *P*=0.049) in the diabetic group [37]. These findings are consistent with the metabolic and hematologic disturbances observed in diabetic patients in contrast with the nondiabetic. Diabetes is associated with accelerated atherosclerosis, widespread endothelial dysfunction resulting in pronounced plaque instability, enhanced platelet aggregation, and clotting response, leading to hypercoagulability [39, 40]. Compared to the nondiabetic, diabetic patients have greater residual plaque burden throughout the reference segment; the intravascular ultrasound study reported the association between greater plaque burden and edge stenosis with DES and BMS [41–43]. This finding may also be true when DCB is used. We observed a numerically higher percentage of post-DCB residual stenosis in diabetic patients.

Contrary to our findings, Jalaluddin reported a significantly higher MACE events (4.3% vs. 0.6%; *P*=0.000), MI (2.6% vs. 0.4%; *P*=0.002) in the diabetic group [37]. A meta-analysis by Sánchez et al. reported that diabetes did not affect the effect of DCB on the TVR [44]. These conflicting results might be related on the one hand to the differences in the inclusion criteria; only patients with ≤2.75 mm vessel diameter were enrolled, and all bailout stenting were excluded from our study. However, Jalaluddin included all patients regardless of the vessel size [37]. Concerning the meta-analysis by Sánchez et al., the heterogeneity of the included trials in terms of the vessel size (<3 mm, <2.8 mm, and ≤2.75 mm) is significant to consider, knowing that the risk of restenosis after PCI is inversely correlated to the treated vessel's size [12, 44, 45]. The enrolled trials included the bailout BMS implantation cases, besides, procedural limitations such as the absence of routine lesion pre dilatation in the PICCOLETO trial, and heterogeneity in the duration of the follow-up among the included trials [44].

## 5. Conclusion

The drug-coated balloon-only treatment achieved lower incidence rates of target lesion failure and major adverse cardiac events in de novo small coronary vessel disease based on our data. We reported significantly higher events rates for TLF and individual TLR in the diabetic group than the nondiabetic. However, numerically higher MACE and individual TVR rates, MI, and cardiac death without statistical significance were observed in the diabetic group compared to the nondiabetic. The presence of diabetes is an independent predictor of TLF and TLR, but not of MACE after 12-month follow-up.

### 5.1. Limitations

First, the absence of angiographic follow-up for a considerable number of our study population and the short duration of the reported follow-up did not assess the long-term outcomes. Second, because of the small caliber of the treated vessels, ischemic symptoms might have been relatively weaker, consecutively our conclusion might have overestimated the performance of DCB.

## Figures and Tables

**Table 1:** Patients' baseline characteristics.

	Overall population (*n* = 1198)	Nondiabetic (*n* = 768)	Diabetic (*n* = 430)	*P* value
*Demographics*				
Sex				
Male	831 (69.3%)	544 (70.8%)	287 (66.7%)	0.141
Female	367 (30.7%)	224 (29.1%)	143 (33.2%)	0.141
Age	60 ± 10.7	59 ± 10.9	60 ± 10.5	0.003^*∗*^
*History and risk factor*				
Family history of CAD	223 (18.6%)	140 (18.2%)	83 (19.3%)	0.643
Current smoker	380 (31.7%)	245 (31.9%)	135 (31.3%)	0.857
Hyperlipidemia	340 (28.3%)	187 (24.3%)	153 (35.5%)	0.001^*∗*^
History of MI	117 (9.7%)	72 (9.3%)	45 (10.4%)	0.542
History of PCI	191 (15.9%)	105 (13.6%)	86 (20%)	0.004^*∗*^
History of CABG	19 (1.5%)	9 (1.1%)	10 (2.3%)	0.125
*Laboratory tests*				
Hemoglobin (g/L)	135 ± 62.4	137 ± 77.4	131 ± 15.2	0.133
Hematocrit (%)	0.80 ± 4	0.94 ± 4.7	0.56 ± 2.4	0.141
Blood glucose (mmol/L)	173 ± 122	145 ± 91.	229 ± 146	0.001^*∗*^
HbA1C (%)	6 ± 3.4	5 ± 0.35	8 ± 5.06	0.001^*∗*^
Urea (mmol/dL)	5 ± 2.9	5 ± 1.8	6 ± 4.2	0.003^*∗*^
Creatinine (*μ*mol/L)	75 ± 38.1	75 ± 34.9	75 ± 43	0.960
eGRF	90 ± 17	90 ± 15.9	89 ± 18.7	0.479
LDL (mmol/dL)	4 ± 64.8	5 ± 82.5	2 ± 0.7	0.424
HDL (mmol/dL)	2 ± 31.7	2 ± 40.3	0.9 ± 0.2	0.405
Albumin (g/L)	10 ± 13.5	10 ± 13.6	10 ± 13.4	0.831
Total cholesterol (mmol/dL)	3 ± 2.5	3 ± 0.9	3 ± 3.9	0.309
Triglyceride (mmol/dL)	1 ± 0.9	1 ± 0.9	1 ± 1.07	0.001^*∗*^
NT-proBNP (pg/ml)	624 ± 1049.2	568 ± 898.5	720 ± 1266.6	0.079
cTnT (ng/L)	11 ± 18.2	11 ± 18.2	12 ± 18.6	0.560
cTnI (ng/L)	1.68 ± 3.8	1 ± 3.5	1 ± 4.3	0.506
*Clinical presentation*				
Hypertension	623 (52.3%)	363 (47.2%)	260 (60.4%)	0.001^*∗*^
LVEF (%)	24 ± 16.6	24 ± 16.7	25 ± 16	0.622
Acute coronary syndrome	802 (66.9%)	515 (67%)	287 (66.7%)	0.912
Renal failure	57 (4.7%)	25 (3.2%)	32 (7.4%)	0.001^*∗*^
Insulin therapy	72 (6%)	0 (0%)	72 (16.7%)	0.001

^*∗*^Statistically significant. CABG: coronary artery bypass graft; CAD: coronary artery disease; CRP: C-reactive protein; MI: myocardial infarction; cTnI: cardiac troponin I; cTnT: cardiac troponin T; eGFR: estimated glomerular filtration rate; HbA1C: glycosylated hemoglobin; HDL: high-density lipoprotein; LDL: low-density lipoprotein; NT-proBNP: N-terminal prohormone brain natriuretic peptide; PCI: percutaneous coronary intervention.

**Table 2:** Baseline lesions characteristics.

	Overall population (*n* = 1198)	Nondiabetic (*n* = 768)	Diabetic (*n* = 430)	*P* value
Bifurcation	418 (34.8%)	259 (33.7%)	159 (36.9%)	0.257
Ostial lesion	193 (16.1%)	121 (15.7%)	72 (16.7%)	0.655
Thrombosis	7 (0.5%)	3 (0.3%)	4 (0.9%)	0.252
CTO	181 (15.1%)	127 (16.5%)	54 (12.5%)	0.65
Diffusion lesion	298 (24.8%)	188 (24.4%)	110 (25.5%)	0.672
Calcified	61 (5%)	42 (5.4%)	19 (4.4%)	0.428
Target vessel				0.112
LAD	505 (42.2%)	314 (40.8%)	191 (44.4%)	0.235
LCX	456 (38.1%)	311 (40.4%)	145 (33.7%)	0.021^*∗*^
RCA	233 (19.4%)	140 (18.2%)	93 (21.6%)	0.154
Graft	4 (0.3%)	3 (0.3%)	1 (0.2%)	0.640
Lesion length	16 ± 8.7	16 ± 8.6	15 ± 8.7	0.644
Treated lesion	1351 (100%)	868 (64.2%)	483 (35.7%)	0.064
1 lesion	1051 (87.7%)	674 (87.7%)	377 (87.6%)	0.965
2 lesions	141 (11.8%)	88 (11.4%)	53 (12.3%)	0.655
3 lesions	6 (0.6%)	6 (0.7%)	0 (0%)	0.066

^*∗*^Statistically significant; CTO: chronic total occlusion; LAD: left anterior descending artery; LCX: left circumflex; RCA: right coronary artery.

**Table 3:** Procedural characteristics.

	Overall population (*n* = 1198)	Nondiabetic (*n* = 768)	Diabetic (*n* = 430)	*P* value
Procedural				0.225
Transradial	1181 (98.5%)	736 (95.8%)	418 (97.2%)	
Transfemoral	44 (3.6%)	32 (4.1%)	12 (2.7%)	
Treated lesion	1351 (100%)	868 (64.2%)	483 (35.7%)	0.064
1 lesion	1051 (87.7%)	674 (87.7%)	377 (87.6%)	
2 lesions	141 (11.8%)	88 (11.4%)	53 (12.3%)	
3 lesions	6 (0.6%)	6 (0.7%)	0 (0%)	
Lesion preparation				
Semicompliant balloon	939 (78.4%)	609 (79.2%)	330 (76.7%)	0.303
Semicompliant balloon diameter (mm)	2 ± 0.2	2 ± 0.2	2 ± 0.2	0.647
Semicompliant balloon pressure (atm)	8.2 ± 3.2	8.2 ± 1.2	8.3 ± 3.6	0.431
Lacrosse NSE®	364 (30.4%)	295 (38.4%)	135 (39.7%)	0.569
Lacrosse NSE® diameter (mm)	2.3 ± 0.2	2.3 ± 0.2	2.3 ± 0.2	0.151
Cutting balloon	130 (10.9%)	81 (10.5%)	49 (11.3%)	0.651
Cutting balloon diameter (mm)	2.5 ± 0.2	2.5 ± 0.2	2.5 ± 0.2	0.536
Scoreflex®	51 (4.3%)	35 (4.5%)	16 (3.7%)	0.492
Scoreflex® diameter (mm)	2.2 ± 0.2	2.2 ± 0.2	2.2 ± 0.2	0.359
NC balloon	207 (17.3%)	137 (17.8%)	70 (16.7%)	0.493
NC balloon diameter (mm)	2.5 ± 0.16	2.5 ± 0.17	2.5 ± 0.16	0.861
NC balloon pressure (atm)	14.3 ± 2.4	14.1 ± 2.3	14.4 ± 3.5	0.347
Rotational atherectomy	26 (2.2%)	21 (2.7%)	5 (1.1%)	0.073
Burr diameter (mm)	1.4 ± 0.1	1.4 ± 0.1	—	—
Dissection after predilation			0.540	
Type A	377 (31.4%)	248 (32.2%)	129 (30%)	0.404
Type B	159 (13.2%)	99 (12.8%)	60 (13.9%)	0.603
Residual stenosis after predilation	21.1 ± 11.2	20.4 ± 10.8	22.2 ± 11.8	0.020^*∗*^
Residual stenosis after DCB	19.7 ± 11.5	19.3 ± 11.6	20.3 ± 11.4	0.145
DCB number				0.463
1 DCB	1119 (93.4%)	716 (93.2%)	403 (93.7%)	
2 DCB	76 (6.3%)	51 (6.6%)	25 (5.8%)	
3 DCB	3 (0.3%)	1 (0.13%)	2 (0.4%)	
Stenosis (%)	88.9 ± 9.4	88.9 ± 9.6	88.8 ± 9.1	0.890
RVD (mm)	2.5 ± 0.7	2.5 ± 0.8	2.5 ± 0.2	0.366
DCB diameter (mm)	2.4 ± 0.2	2.4 ± 0.2	2.4 ± 0.2	0.629
DCB length (mm)	23.2 ± 9.3	23.3 ± 9.2	23 ± 9.4	0.660
DCB inflation pressure (atm)	8.2 ± 3.2	8.1 ± 1.4	8.3 ± 5.1	0.416
DCB inflation time (s)	60 ± 4.5	60.1 ± 4.9	59.9 ± 3.7	0.474
Dissection after DCB deployment			0.773	
Type A	338 (28.2%)	218 (28.3%)	120 (27.9%)	0.860
Type B	172 (14.4%)	109 (14.1%)	63 (14.6%)	0.835

^*∗*^Statistically significant; DCB: drug-coated balloon; NC: noncompliant; RVD: reference vessel diameter.

**Table 4:** One-year outcomes.

Study endpoints	Overall population (*n* = 1198)	Nondiabetic (*n* = 768)	Diabetic (*n* = 430)	*P* value
TLF	28 (2.3%)	11 (1.4%)	17 (3.9%)	0.006^*∗*^
TLR	13 (1%)	4 (0.5%)	9 (2%)	0.014^*∗*^
TVMI	4 (0.3%)	1 (0.1%)	3 (0.6%)	0.110
Cardiac death	11 (0.9%)	6 (0.7%)	5 (1.1%)	0.513
MACE	40 (3.3%)	21 (2.7%)	19 (4.4%)	0.120
Myocardial infarction	4 (0.3%)	1 (0.1%)	3 (0.6%)	0.110
TVR	18 (1.5%)	8 (1%)	10 (2.3%)	0.080
All-cause death	18 (1.5%)	12 (1.5%)	6 (1.3%)	0.820
Others				
CABG + PCI	56 (4.6%)	33 (4.2%)	23 (5.3%)	0.408

*Angiographic follow-up at 12 months*				
	Overall population (*n* = 535)	Nondiabetic (*n* = 349)	Diabetic (*n* = 186)	*P* value

Reference vessel diameter (mm)	2.5 ± 0.7	2.5 ± 0.8	2.5 ± 0.2	0.629
Minimum lumen diameter before angioplasty (mm)	0.35 ± 1.87	0.39 ± 2.34	0.28 ± 0.23	0.315
Minimum lumen diameter after angioplasty (mm)	2.06 ± 0.35	2.07 ± 2.35	2.04 ± 2.35	0.136
Acute gain	1.79 ± 0.41	1.8 ± 0.41	1.77 ± 0.42	0.154
Minimum lumen diameter 12 months after angioplasty (mm)	1.97 ± 0.44	2 ± 0.39	1.89 ± 0.53	0.007^*∗*^
Late lumen loss (mm)	0.12 ± 0.30	0.09 ± 0.25	0.17 ± 0.37	0.003^*∗*^
Binary restenosis, *n* (%)	13 (2.4%)	4 (1.1%)	9 (4.8%)	0.008^*∗*^

^*∗*^Statistically significant. CABG: coronary artery bypass graft; MACE: major adverse cardiac events; PCI: percutaneous coronary intervention; TLF: target lesion failure; TLR: target lesion revascularization; TVMI: target vessel myocardial infarction; TVR: target vessel revascularization.

**Table 5:** Cox proportional hazard multivariate analysis for TLF and TLR.

		*P* value	HR	95% CI	
				Lower	Upper
TLR	Age	0.841	1.006	0.951	1.064
	Sex	0.597	1.610	0.276	9.389
	Hypertension	0.135	2.741	0.731	10.275
	Smoking	0.082	3.144	0.865	11.423
	Hyperlipidemia	0.579	1.389	0.435	4.436
	Diabetes	0.033^*∗*^	3.698	1.112	12.298
	Renal insufficiency	0.955	1.064	0.124	9.144

TLF	Age	0.998	1.000	0.963	1.038
	Sex	0.652	1.238	0.490	3.127
	Hypertension	0.288	1.540	0.694	3.415
	Smoking	0.807	1.121	0.448	2.808
	Hyperlipidemia	0.136	1.797	0.831	3.889
	Diabetes	0.011^*∗*^	2.712	1.254	5.864
	Renal insufficiency	0.601	0.577	0.074	4.533

*Notes*. Cox proportional hazard multivariate analysis was performed using age, gender, hypertension, smoking, hyperlipidemia, diabetes, and renal insufficiency. ^*∗*^Statistically significant at *P* ≤ 0.05. CI: confidence interval; HR: hazard ratio.

## Data Availability

The datasets used and/or analyzed during the current study are available from the corresponding author upon reasonable request.
